# P-TEFb Kinase Activity Is Essential for Global Transcription, Resumption of Meiosis and Embryonic Genome Activation in Pig

**DOI:** 10.1371/journal.pone.0152254

**Published:** 2016-03-24

**Authors:** Reza K. Oqani, Tao Lin, Jae Eun Lee, Ki Myung Choi, Hyun Young Shin, Dong Il Jin

**Affiliations:** 1 Department of Animal Science & Biotechnology, Research Center for Transgenic Cloned Pigs, Chungnam National University, Daejeon, 305–764, Republic of Korea; 2 Optipharm Inc, Cheongju, Chungbuk, 361–954, Republic of Korea; University of Hyderabad, INDIA

## Abstract

Positive transcription elongation factor b (P-TEFb) is a RNA polymerase II carboxyl-terminal domain (Pol II CTD) kinase that phosphorylates Ser2 of the CTD and promotes the elongation phase of transcription. Despite the fact that P-TEFb has role in many cellular processes, the role of this kinase complex remains to be understood in mammalian early developmental events. In this study, using immunocytochemical analyses, we found that the P-TEFb components, CDK9, Cyclin T1 and Cyclin T2 were localized to nuclear speckles, as well as in nucleolar-like bodies in pig germinal vesicle oocytes. Using nascent RNA labeling and small molecule inhibitors, we showed that inhibition of CDK9 activity abolished the transcription of GV oocytes globally. Moreover, using fluorescence in situ hybridization, in absence of CDK9 kinase activity the production of ribosomal RNAs was impaired. We also presented the evidences indicating that P-TEFb kinase activity is essential for resumption of oocyte meiosis and embryo development. Treatment with CDK9 inhibitors resulted in germinal vesicle arrest in maturing oocytes *in vitro*. Inhibition of CDK9 kinase activity did not interfere with *in vitro* fertilization and pronuclear formation. However, when *in vitro* produced zygotes were treated with CDK9 inhibitors, their development beyond the 4-cell stage was impaired. In these embryos, inhibition of CDK9 abrogated global transcriptional activity and rRNA production. Collectively, our data suggested that P-TEFb kinase activity is crucial for oocyte maturation, embryo development and regulation of RNA transcription in pig.

## Introduction

Embryonic genome activation is a highly regulated process by which an embryo begins to produce its own gene products from its newly formed genome. Before the embryonic genome is activated, the embryo is transcriptionally inactive and is dependent on the factors already provided by the oocyte. These factors (mainly proteins and mRNAs) are produced during the course of oocyte growth until the oocyte becomes competent for resumption of meiosis. Before the oocytes become competent to maturation process, the oocyte genome undergoes changes in genome architecture and function which prepare an epigenetic context for the developmental regulation of the global gene expression [1]. Along with the changes in epigenetic landscape, oocytes arrested at the prophase of the first meiotic division undergo an intensive change in their chromatic shape. As oocytes grow, their chromatin configuration changes from an open chromatin dispersed throughout the nucleus (germinal vesicle) to a ring-shaped condensed chromatin surrounding the massive nucleolus-like body at the final phase of growth [2]. This change results in a transcriptionally silenced chromatin [3]. Similar to human nuclei [4], tens of discrete transcription sites scattered throughout the GV can be detected under a confocal microscope. By transition from NSN (non-surrounded nucleolus) to SN (surrounded nucleolus) configuration, however, the number and fluorescence intensity of transcription sites declines and in SN oocytes, become undetectable. Superimposed on this change in chromatin architecture is change in transcriptional activity in oocytes nuclei. In mice, it has been shown that in NSN oocytes, BrUTP incorporation into nascent RNAs is relatively robust and is both RNA polymerase I (Pol I)- and RNA polymerase II (Pol II)-dependent, while SN oocytes are transcriptionally inactive [5]. We also have shown that pig GV oocytes follow a very similar pattern [6]. Labeling of nascent RNA with another halogenated nucleotide, 5-fluorouridine (FU), showed that in pig NSN and pNSN oocytes, the level of RNA synthesis is much higher than that of pSN oocytes; and SN oocytes are absolutely transcriptionally silenced.

Pol I mainly synthesizes ribosomal RNAs, while Pol II is responsible for mRNAs and snRNAs production. Although the regulation of rRNA synthesis is well studied in GV oocytes, the mechanism(s) regulating Pol II-dependent transcription is less understood in mammalian oocytes. Pol I and its related transcription factors such as UBF and SL1, are located specifically in the nucleolus. The nucleolus is a prominent sub-nuclear structure that is responsible for the biogenesis of ribosome subunits, 18S, 5.8S and 28S rRNAs. Electron microscopy has permitted researchers to discern three main nucleolar compartments: the fibrillar centers (FCs), the dense fibrillar component (DFC), and the granular component (GC) [7]. Pol I is the enzyme complex responsible for the initial transcription of rDNA genes that are organized in arrays of repeats called nucleolar organizer regions (NORs) [8, 9]. Pol I subunits are enriched in the FCs and implement rDNA transcription at the border of the FC and DFC regions [10–13]. Proteins responsible for early rRNA processing like nucleolin and fibrillarin accumulate in the DFC, whereas nucleophosmin, involved in late rRNA processing, is localized in the GC [14–16].

In few studies, the presence and the phosphorylation status of Pol II in mammalian GV oocytes have been investigated [17–19]. Pol II is responsible for synthesis of mRNAs and some non-coding RNAs. This enzyme complex consists of 12 subunits among them the largest one (Rpb1) contains a very unique carboxyl-terminal domain (Pol II CTD) which composed of multiple heptapeptide motif, YSPTSPS. Phosphorylations of serine residues of this motif, which repeats itself 52 times in mammalian cells, regulates the function of the Pol II complex as phosphorylation of Ser5 residues by TFIIH (CDK7/Cyclin H/Mat1) is correlated with transcription initiation, and phosphorylation of Ser2 residues by P-TEFb (CDK9/Cyclin T) regulates the transition from initiation to productive elongation. Studies show that Pol II is present and functional in growing oocytes and exhibit lower accumulation and activity as the oocytes approach to their end of the growth phase. In fact, in fully-grown oocytes, active forms of Pol II (phosphorylated CTD) become almost undetectable when analyzed by Western blotting or immunocytochemistry [18, 19]. This phenomenon is concomitant with gradual shut-down of transcription in oocytes before GVBD.

Gene expression is highly regulated at transcription phase. Pol II function is controlled in multiple steps mainly via phosphorylation and dephosphorylation of its CTD [20–22]. The phosphorylation of Pol II CTD on Ser2 promotes the transition from initiation to elongation phase of transcription. Also, negative transcription factors DSIF and NELF must be phosphorylated by P-TEFb [23]. Positive transcription elongation factor, P-TEFb, consists of cyclin-dependent kinase 9 (CDK9) and a cyclin regulatory partner (Cyclin T). Expression of most protein coding genes is negatively affected by inhibiting P-TEFb kinase activity by Flavopiridol [24]. A recent study has shown that P-TEFb inhibition by 300 nM Flavopiridol decreases the expression of 95 percent of genes in mouse embryonic stem cells [25].

P-TEFb contributes to multiple cellular functions. For instance, multiple steps of gene expression are influenced by P-TEFb. Beside its crucial role in elongation phase of transcription, P-TEFb function regulates co-transcriptional control of mRNA processing, export as well as mRNA translation in the cytoplasm [26]. Expression of many genes in mammalian cells is regulated by the recruitment of P-TEFb via Brd4, a bromodomain protein that binds highly acetylated chromatin [27]. It has been shown that Brd4 associates with mitotic chromosomes and possibly plays role in the mitotic bookmarking of active genes. Studies have shown that at the late mitosis (telophase), both CDK9 and CyclinT1 are recruited to chromosomes in a Brd4-dependent manner [28]. Also it has been shown that although the expression level of P-TEFb components is not change significantly through cell cycle, the level of Brd4-bound P-TEFb increases from the late mitosis to early G1 therein, P-TEFb is recruited to telophse chromosomes and initiates early transcription of key genes for G1 progression [29]. P-TEFb also has additional contribution to the cell cycle progression. For instance, CDK9/Cyclin T2 (but not Cyclin T1), interacts with retinoblastoma protein (pRB) [30]. CDK9 also phophorylates p53 on serine residues S33, S315, and S392 [31]. As such, knockdown of CDK9 by RNAi leads to G1 arrest of cells, suggesting a role of P-TEFb in G1/S transition [32].

The role of P-TEFb in development has been poorly understood. Genetic inactivation of cit.1.1 or cit.1.2, the closely related *C*. *elegans* cyclin T genes by RNAi had no dramatic change in its development. But when both cyclins were genetically inactivated simultaneously, embryos died at ~100-cell stage. Same phenotype was observed when CDK9 was knocked down by RNAi, indicating the crucial role of P-TEFb in early embryonic development [33]. The first attempt to address the role of P-TEFb in fly’s early embryogenesis has shown similar results. Down regulation of maternal CDK9 or Cyclin T, leads to early embryonic lethality [34]. In mice, unlike *C*. *elegans* in which cyclin Ts are redundant, Cyclin T2 knockout results in early embryonic lethality [35].

Despite the critical role of P-TEFb in many cellular functions, there is no report to address the necessity of CDK9 or its major partner Cyclin T1 in mammalian oocyte development or embryogenesis. We have previously shown that inhibition of CDK9 kinase activity leads to 2-cell stage arrest in mouse embryos. Here, for the first time, we study the presence and functionality of P-TEFb in pig preovulatory oocytes and early embryos. We show that inhibition of CDK9 by its specific inhibitors, arrests oocytes at GV stage, and embryos at 4-cell stage. In addition, we show that P-TEFb beside its known function in general transcription, may play role in ribosomal RNA transcription.

## Materials and Methods

### Ethics and Chemicals

All animal care and use procedures were approved by the Institutional Animal Care and Use Committee of Chungnam National University. Flavopiridol (F3055), α-amanitin (A2263), ActD (A1410), DRB (D1916), and 5-Fluorouridine (F5130) were purchased from Sigma and dissolved in DMSO or sterile double-distilled water to form 0.5 mM, 1mM, 100 μM, 100 mM and 0.25 M stock solutions, respectively. Dinaciclib (S2768) and JNJ-7706621 (S1249) were purchased from Selleck Chemicals and dissolved in DMSO to form 0.5 mM stock solutions. CDK9 Inhibitor II/CAN508 (sc-203326) was purchased from Santa Cruz and dissolved in DMSO to form 5 mM stock solution. Rabbit polyclonal antibodies against CDK9 (C-20), Cyclin T1 (H-245) and Cdc2 p34 (F-5), and mouse monoclonal antibodies against Fibrillarin (G-8), UBF (F-9), Lamin A/C (636) and p-Cdc2 p34 (Thr 14/Tyr 15) were purchased from Santa Cruz and diluted 1:50. Rabbit polyclonal antibody against phospho-CDK9 on Thr186 (#2549) was purchased from Cell Signaling and diluted 1:25. Rabbit polyclonal antibody against Cyclin T2 (ab96133) was from Abcam and diluted 1:50. Monoclonal antibody against Pol II CTD phospho S2 (H5) was purchased from Covance and diluted 1:50. Anti-SC35 mouse monoclonal antibody (Sigma, S4045) and anti-BrdU monoclonal antibody (Sigma, B2531) were diluted 1:5000 and 1:200 respectively. Secondary antibodies were conjugated with FITC or Texas Red and purchased from Santa Cruz.

### Collection and culture of porcine oocytes and production of IVF and parthenogenetic embryos *in vitro*

Porcine ovaries were collected from prepubertal gilts at a local abattoir (NH Livestock Cooperation Association, N36.201687 E127.084476, Nonsan City, Chungnam Province, Korea) where we had acquired permission, and transported to the laboratory at 30–35°C. They were washed three times with warm PBS containing 100 IU/mL penicillin and 50μg/mL streptomycin and stored in a water bath at 37°C until use. COCs were aspirated from ovarian follicles, 2–6 mm in diameter, and washed three times in TL-HEPES containing 0.1% (w/v) PVA. They were then either subjected to the experiment or were transferred to 500 μL of maturation medium, which had been covered with mineral oil, in a four-well multidish (Nunc, Roskide, Denmark) and incubated for 44 h at 38.5°C in an atmosphere of 5% CO_2_ at maximum humidity. Medium used for *in vitro* maturation of COCs was bovine serum albumin (BSA)-free M199 supplemented with 10% (v/v) porcine follicular fluid (FF), 0.57 mM l-cysteine, 2% (v/v) basal medium Eagle amino acids, 1% (v/v) minimum essential medium non-essential amino acids, 0.5 μg/mL LH, 0.5 μg/mL FSH, 10 ng/ml epidermal growth factor, 75 μg/mL penicillin G and 50 μg/mL streptomycin. After 22 h in maturation culture, the COCs were washed three times and transferred to 500 μL of basic medium without hormone for an additional 22 h of culture.

For *in vitro* fertilization, after *in vitro* maturation, cumulus cells were removed by repeated pipetting in TL-HEPES supplemented with 0.1% PVA and 0.3% hyaluronidase. Only oocytes with the first polar body extruded were collected. Oocytes were washed 2 times with mTBM containing 0.1% BSA as IVF medium and kept in 100 μl drops of mTBA/BSA under pre-warmed mineral oil until insemination. Motile sperm from fresh semen were washed two times via centrifugation at 1,200 rpm for 3 min in mTBM and incubated for 5 min at 38.5°C for swim-up. Sperm were collected from the surface and added to oocyte-containing IVF droplets. Twenty-five to 30 oocytes were inseminated in each drop. Insemination lasted for 6 h. Oocytes were removed from fertilization drops, washed three times, and cultured in PZM-3 medium for 7 days.

For parthenogenetic activation, following *in vitro* maturation and denuding, oocytes were transferred to the activation solution, consisting of 0.3 M d-mannitol, 0.1 mM MgSO_4_, 0.05 mM CaCl_2_, and 0.01% PVA, and washed three times. The oocytes were then stimulated with a 1.5 kV/cm direct current-pulse for 100 μs using a BTX Elector-Cell Manipulator 2001 (BTX, San Diego, CA, USA). After activation, eggs were washed and transferred into 500 μL of culture media covered with mineral oil in a four-well multidish. IVC medium was prepared in porcine zygote medium-3 (PZM-3). The culture environment was 5% CO_2_ and 20% O_2_ at 38.5°C for 7 days.

### Western blotting

COCs were denuded and GV oocytes (50 per sample) were washed three times in PVA–PBS, and then resuspended in extraction buffer (PRO-PREP; Intron Biotechnology, Seoung, Korea). The extracted proteins were separated by 10% (w/v) SDS–PAGE using a Bio-Rad apparatus (Bio-Rad) and then electrophoretically transferred to PVDF membranes, employing a Bio-Rad Mini Trans-Blot Cell. The membrane was blocked with 5% (w/v) skim milk and 0.5% (v/v) Tween-20 in Tris-buffered saline and subsequently exposed to primary antibody directed against CDK9 at 1:1000 dilution. The antibody solution was prepared in Tris-buffered saline, containing 5% (w/v) nonfat dry milk powder and 0.1% (v/v) Tween-20. The membrane was next washed in Tris-buffered saline with 0.5% (v/v) Tween-20 for 15 min and antibody–antigen complexes were detected using anti-rabbit IgG peroxidase conjugates (Abcam, ab6721, Cambridge, MA, USA), followed by employment of an ECL detection kit (Amersham Bioscience).

### Immunostaining and in situ run-on transcription

For immunofluorescence staining, oocytes or embryos were washed twice in 0.1% (w/v) PVA in PBS and fixed in 2% (v/v) formaldehyde in PBS for 15 minutes at room temperature. Next, oocytes were permeabilized for 30 minutes in 0.5% (v/v) Triton X-100 in PBS, washed for 10 minutes in 100 mM glycine in PBS (to inactivate free aldehyde groups), and nonspecific binding sites were blocked with 3% (w/v) BSA for 20 minutes, followed by 5 minutes in PBG [PBS containing 0.5% (w/v) BSA and 0.1% (w/v) gelatin from the skin of cold-water fish (Sigma)]. Incubations with primary antibodies proceeded in PBG for 16 h at 4°C. Cells were subsequently washed four times, for 5 min. each time, in PBG and incubated with the appropriate secondary antibodies for 1 h in PBG at room temperature. Next, the cells were washed twice, for 5 minutes each time, in PBG and twice for 5 minutes each time in PBS. For microscopic observation, embryos were deposited on slides and mounted under coverslips using Vectashield mounting medium containing DAPI (Vector Laboratories). In situ run-on transcription was performed as described earlier [9], with some modifications. Briefly, oocytes or embryos were rinsed with PBS/PVA, followed by incubation in 5mM 5-FU for indicated times. Immunolabeling was achieved by overnight incubation with mouse monoclonal anti-BrdU antibody followed by washing and further incubation for 1 h with Texas Red-conjugated mouse IgG.

### Fluorescence in situ hybridization

Oocytes or embryos were fixed in 2% formaldehyde for 15 min and then washed twice in PBS/PVA for 5 min. at room temperature. Cells were then permeabilized with 1% Triton X-100 in PBS for 30 min. Then, cells were washed in 2X saline-sodium citrate (SSC) plus 50% formamide for 5 min. at room temperature. Hybridizations occurred over night at 38.5°C in hybridization buffer consisting of: 10% dextran sulfate, 2mM vanadyl ribonucleoside complex, 0.02% BSA, 40 μg tRNA, and 10 ng of RNA probe in 2X SSC plus 50% formamide. Then, cells were washed with pre-warmed 2X SSC/50% formamide at 37°C for 30 min. Finally, cells were washed twice in PBS/PVA and mounted in Vectashield mounting medium containing DAPI. The sequences of the probes corresponding to pig 5’ETS rRNA (Accession no: L31782) and 28S rRNA (Accession no: 4W24_5) were:

5’ETS antisense: Cy3-gccagagaccggaggagacaccgcagcacgggcc; 5’ETS sense: Cy3-ggcccgtgctgcggtgtctcctccggtctctggc. 28S antisense: FAM-ccccacccgtttacctcttaacggtttcacgccc; 28S sense: FAM-gggcgtgaaaccgttaagaggtaaacgggtgggg. The probes were produced by Bioneer, Inc. Korea.

### Confocal microscopy and fluorescence intensity measurement

Images were captured using a Zeiss scanning laser confocal microscope running Zeiss LSM Image Browser software. Serial optical sections (the Z-series) were collected at 0.5 μm intervals. The Z-stacks were captured and images depicting staining patterns and intensities of all nuclear areas were generated. All oocyte and embryo samples were prepared and processed simultaneously prior to fluorescence intensity measurements. The laser power was adjusted to ensure that signal intensity was below saturation for the specimen that displayed the highest intensity and all images were next scanned at that laser power.

## Results

### P-TEFb components are present in fully-grown oocytes

**Fig 1:**
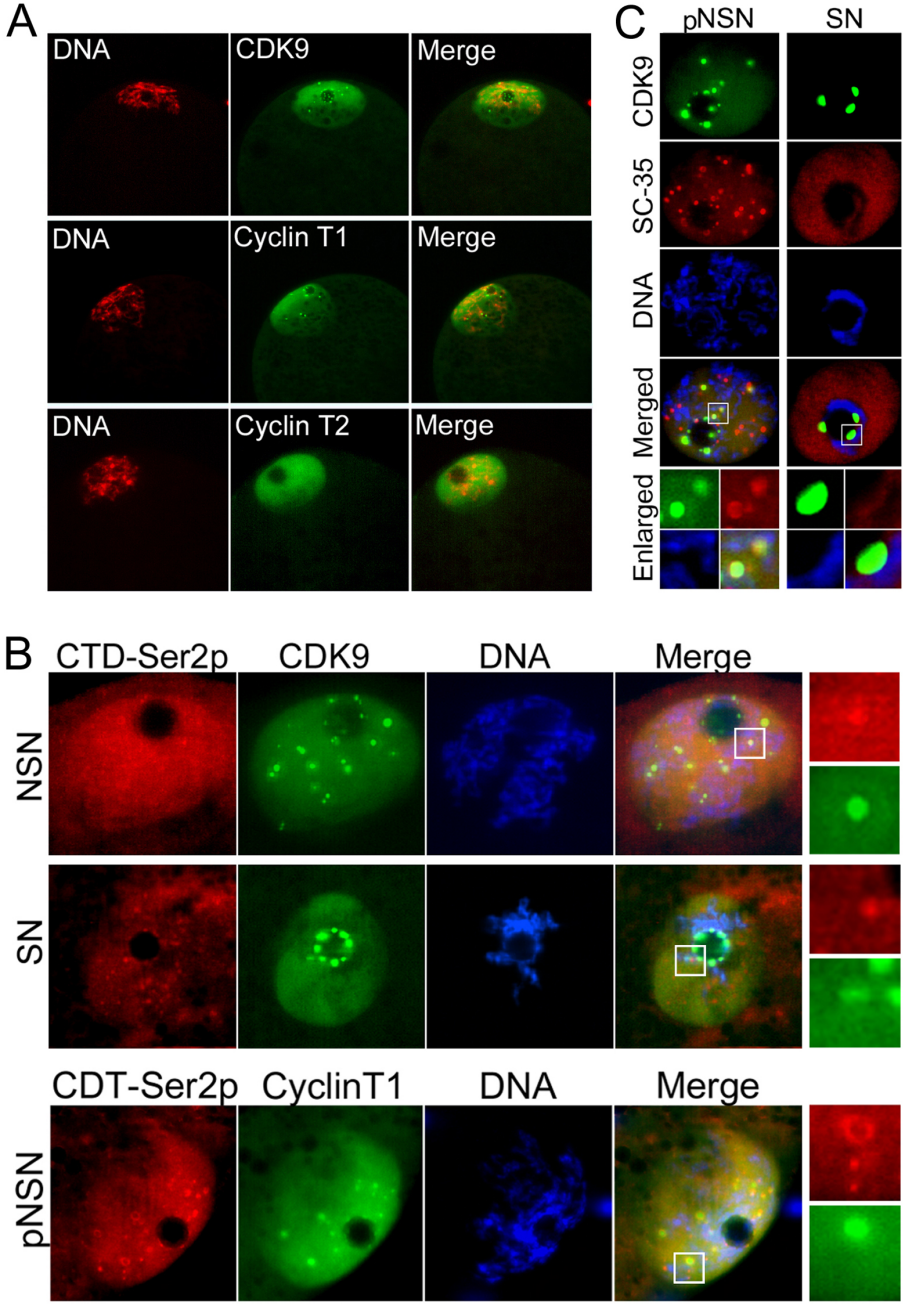
Subcellular localization of P-TEFb components in GV of fully grown pig oocytes. **A)** CDK9, Cyclin T1 and Cyclin T2 are present and located predominantly in the nuclei of pig GV oocytes. CDK9 and Cyclin T1 also show speckles in GV oocytes. Rabbit polyclonal antibodies were used for immunostaining. **B)** CDK9 and Cyclin T1 speckle-like structures are colocalized with Pol II CTD Ser2p in GV of NSN and pNSN oocytes. In SN oocytes, CDK9 tends to accumulate at the periphery of the NLB and also dissociate from Pol II CTD Ser2p speckles. **C)** CDK9 also colocalizes with the splicing speckle marker SC-35 protein (red) in GV of pNSN oocytes. In transcriptionally inactive SN oocytes, SC-35 is dispersed throughout the nucleoplasm but CDK9 aggregates around the NLB. SC-35 is immunostained with a mouse monoclonal antibody. DNA is counterstained with DAPI.

The presence and localization of P-TEFb components have never before been addressed in porcine oocytes. Initially, using specific antibodies, we observed that CDK9, Cyclin T1 and Cyclin T2 are present and dominate the GVs of oocytes obtained from pig antral follicles (Fig 1A). Similar to what we observed in mice oocytes (our unpublished data), CDK9 and Cyclin T1 also exhibited large round bodies scattered throughout the GVs of pig oocytes with open chromatin (NSN and partly NSN). Also we found that phosphorylated Pol II on ser2 of the CTD (CTD-Ser2p) but not CTD-Ser5p or CTD-Unp exhibited several nuclear speckles in pig NSN and pNSN oocytes (data not shown). Therefore, we co-immunostained CDK9 or Cyclin T1 with CTD-Ser2p and found that in NSN both P-TEFb components were either co-localized with or juxtaposed to CDT-Ser2p speckles (Fig 1B). However, there were some Ser2p accumulations not colocalized with P-TEFb components. In SN oocytes, it seemed that CDK9 and Cyclin T1 were dissociated from the nuclear speckles and accumulated around the nucleolar-like bodies (NLBs). Co-immunostaining with splicing speckle marker, SC-35, revealed that CDK9 were partially colocalized with SC-35 in pNSN oocytes (Fig 1C). In other words, some CDK9 speckles did not completely coincide with SC-35 but were juxtaposed to the factor. This suggests that at least in GV oocytes a population of P-TEFb accumulates in nuclear bodies other than mRNA splicing speckles. In SN oocytes, although splicing speckles marked by SC-35 were dispersed throughout the nucleoplasm, CDK9 accumulated strictly around the NLBs.

### P-TEFb is associated with NLB in oocytes

**Fig 2:**
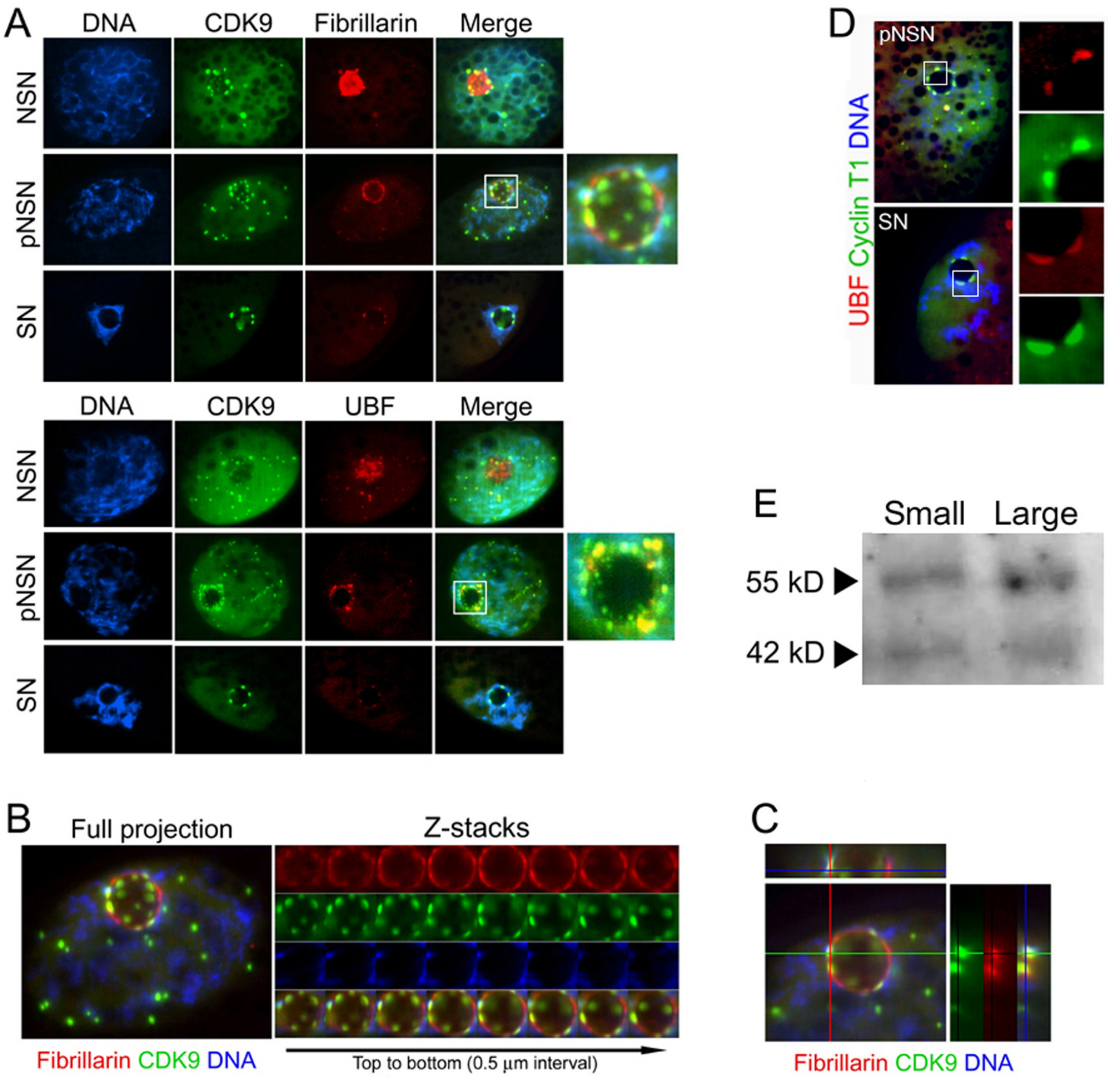
P-TEFb association with nucleolar proteins in the germinal vesicle oocytes at different stages of growth. **A)** CDK9 is colocalized with the DFC component, fibrillarin, only at the periphery of the NLB in NSN oocytes. In pNSN oocytes, nucleolar structure is changed and fibrillarin and other nucleolar components move to the periphery of the NLB. In this stage, CDK9 shows partial colocalization with fibrillarin (inset). In SN oocytes, fibrillarin is mostly gone, but CDK9 remains bound to the nucleolar structure. At the periphery of the NLB, CDK9 shows more colocalization with the FC component, UBF, in pNSN (inset). **B)** The same pNSN oocytes as in A was analyzed further. Eight optical sections, Z-stacks with 0.5 μm, were taken by confocal microscope confirming the colocalization of CDK9 and fibrillarin at the periphery of NLB. **C)** X and Y axes of a single focal plane of the same image as B focusing on an area corresponding to the NLB periphery, indicating the spatially colocalization of CDK9 and fibrillarin in that area. **D)** Cyclin T1 also shows colocalization with nucleolar factor, UBF, in pNSN and pSN oocytes. DNA counterstained with DAPI. **E)** COCs were collected separately from small or large follicles and then were denuded and subjected to Western blotting. The same anti-CDK9 antibody as used for immunostaining was used for Western blot. The bands correspond to two CDK9 isoforms, 42 kD and 55 kD. Fifty oocytes were collected for each group.

To our knowledge, the nucleolar association of P-TEFb components had not been addressed previously in oocytes or embryos and we were curious to see the extent, if any, of CDK9 or Cyclin T1's spatial connection to the nucleolus in pig GV oocytes. Co-immunostaining of CDK9 with nucleolar markers, fibrillarin revealed that in NSN oocytes, fibrillarin was strongly accumulated inside the NLB and some of the CDK9 accumulations closed to NLB were colocalized with marginal nucleolar fibrillarin (Fig 2A). In pNSN oocytes, the nucleolar accumulation of fibrillarin decreased and but still co-localized partly with CDK9. Also in nucleoplasm, fibrillarin showed less intense small bodies co-localized with CDK9 accumulations. The same pattern of distributions and co-localizations were observed when CDK9 was co-immunostained with another nucleolar factor, upstream binding factor (UBF). Both UBF and fibrillarin were predominant in the nucleolar core when oocytes exhibited a roughly dispersed chromatin configuration (NSN) throughout the nucleoplasm. In these cells, CDK9 was attached to the nucleolar components at the periphery of the nucleolus (Fig 2A). By changing the chromatin to pNSN configuration, UBF and fibrillarin moved toward the nucleolar periphery and were more associated with CDK9. Analyses of optical sectioning confirmed that at least some CDK9 accumulations at the periphery of the NLB colocalized strongly with fibrillarin (Fig 2B and 2C). This partial colocalization was also observed with another DFC component, nucleolin (data not shown). The signal intensity of the nucleolar components decreased dramatically in SN oocytes whereas CDK9 exhibited large hemisphere bodies attached to the NLB. Cyclin T1 also displayed a very similar distribution in pig oocytes (Fig 2D). These observations suggested that P-TEFb could be engaged with rRNA transcription factors in transcriptionally active oocytes.

We and others have previously shown that NSN oocytes are mostly found in COCs obtained from small follicles. Also, the population of SN oocytes is higher when COCs are extracted from large follicles [5, 6]. We were curious if the level of CDK9 changes during the growth phase of the oocytes. To this, we collected oocytes separately from small or large follicles and detected CDK9 by immunobloting using the same antibody. Western blot analysis showed that in both population, splicing isoforms of CDK9, 42 kD and 55 kD, are expressed. The level of 42 kD isoform did not change in either population but 55 kD showed a slightly more intensity in oocytes from large follicles (Fig 2E).

### CDK9 is connected to transcription sites and its kinase activity is necessary for global transcription

**Fig 3:**
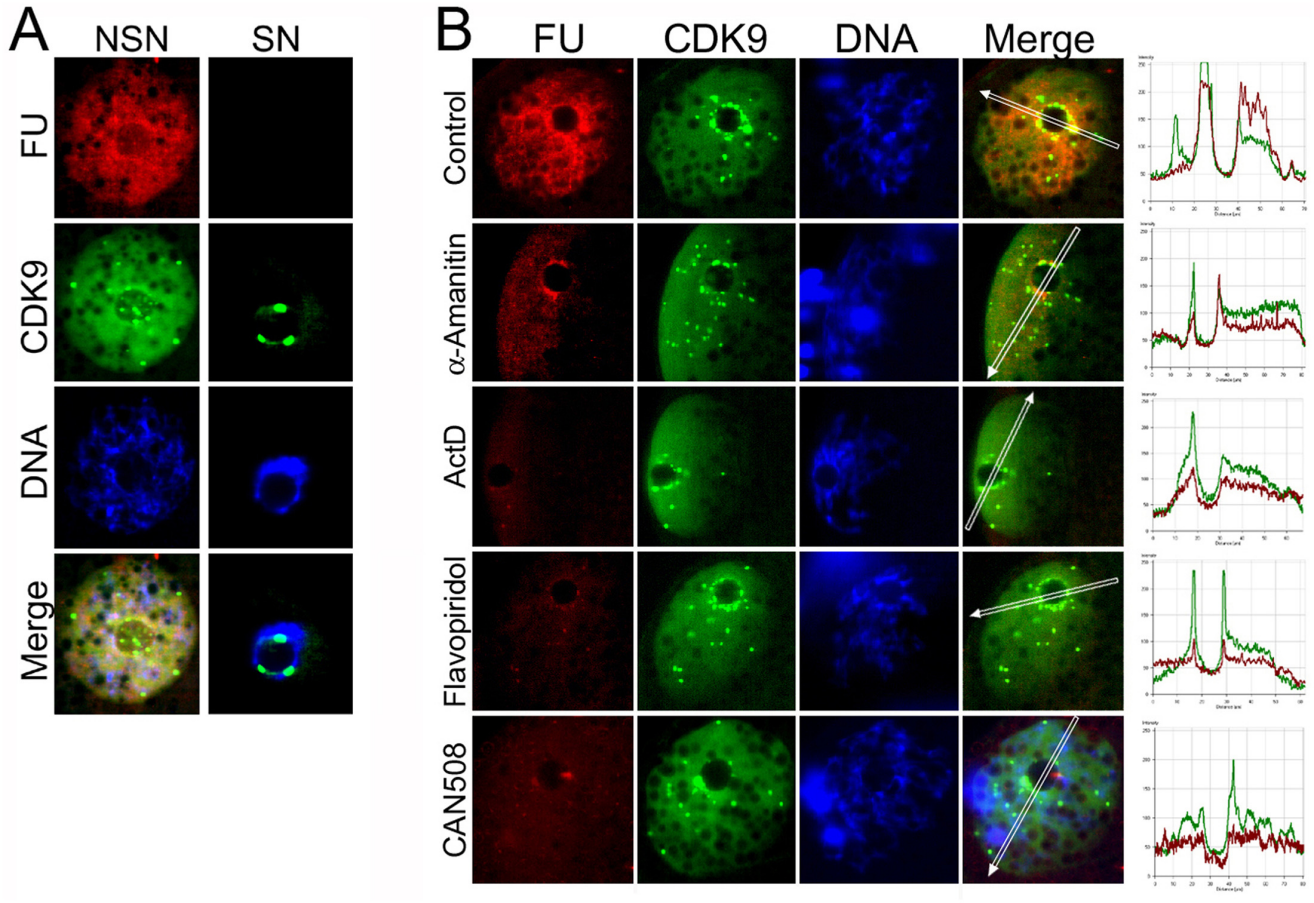
Effect of CDK9 inhibition on transcriptional activity of GV oocytes. **A)** COCs were collected from different size of follicles and immediately cultured in a complete maturation medium in presence of 5 mM FU for 1 hour. Then, COCs were denuded and oocytes were fixed and subjected to immunostaining. In NSN oocytes in which CDK9 is distributed throughout the GV, transcriptional activity is at the highest level. In SN oocytes, CDK9 accumulates around the NLB and nascent RNAs become undetectable. **B)** COCs were subjected or not to α-amanitin, actinomycin D (ActD), or Flavopiridol for 1 hour. Treatment with α-amanitin started 1 hour ahead of the other compounds. At the same time, nascent transcripts were labeled with FU. Then, COCs were denuded and oocytes were fixed and subjected to immunostaining. In α-amanitin-treated oocytes, nucleoplasmic transcription decreased dramatically, but a rim of transcripts remained around the nucleolus. In oocytes treated with ActD, no transcriptional activity was detected. In Flavopiridol-treated oocytes, similarly, almost all the RNA transcription was abolished. Similar result was obtained when oocytes treated with CDK9 Inhibitor II (CAN508).

The association of CDK9 to the nucleolar structures prompted us to consider the existence of a functional relation between CDK9 and the nucleolus. To study this, we selected GV oocytes in their active transcription state, NSN configuration. Regarding the sensitivity of pig oocytes to permeabilization and microinjection, we employed a cell-permeable modified RNA precursor, 5-fluorouridine (FU), to identify transcription sites and changes occurring during pig oocyte growth. Our previous experiments showed that FU can be successfully incorporated into both nascent RNAs by simple incubation of oocytes in FU-containing culture medium followed by immunostaining. Moreover, FU incorporation appears sensitive to both RNA synthesis inhibitors and activators [6]. First, the transcriptional activities of the oocytes were analyzed by confocal microscopy. About 89% of NSN and 79% of pNSN of non-treated oocytes showed sharp and clear nascent RNA labeling. pSN and SN oocytes showed no transcription and were excluded from the analyses (data not shown). Then, we labeled nascent RNAs in oocytes from small or large follicles and co-immunostained them with CDK9 to find a possible association between dynamic changes in CDK9 distribution through oocyte growth and the level transcriptional activity in these cells (Fig 3A). This analysis showed that in NSN oocytes in which CDK9 is dispersed throughout the nucleoplasm, the transcriptional activity was at highest level. When chromatin configuration changed to SN state and CDK9 accumulated around the NLB, the transcriptional activity of the oocytes became undetectable.

To obtain a better understanding about the CDK9’s role in oocyte transcription, we treated GV oocytes in their transcriptionally active state (NSN) with CDK9 specific inhibitors Flavopiridol and CDK9 Inhibitor II (CAN508). When COCs were treated with either compound for 1 hour and labeled with FU, no transcriptional activity was observed in NSN or in pNSN oocytes. When analyzed carefully under a confocal microscope, we observed that inhibition of CDK9 activity resulted in decline in transcription almost globally which differed with the situation in α-amanitin-treated oocytes reported previously [6]. To obtain a better illustration of the effect of CDK9 inhibition, we compared the level and the distribution pattern of nascent RNAs in GV oocytes in which CDK9 activity was inhibited with that of oocytes treated with other transcription inhibitors, α-amanitin and actinomycin D (ActD), with different mechanism of action. In low concentrations (up to 10 μM), α-amanitin inhibits only Pol II activity. ActD, on the other hand, inhibits all the RNA polymerases when is applied in high concentrations (100 nM and higher). Initially, we observed that in the cases of Flavopiridol and ActD the signals emitted from the FU labeling declined almost to the level of background, thus we included CDK9 immunostaining to the experiment to see whether or not inhibition of transcription also decreases the CDK9’s nuclear situation (Fig 3B). In untreated oocytes, the highest level of transcriptional activity was observed around the NLBs. Fluorescence intensity measurements revealed that this pick of transcripts was coincided with CDK9 accumulations around the NLB. Treatment with either CDK9 inhibitors inhibited all the nucleoplasmic transcription as well as almost all the transcriptional activity in NLB periphery but CDK9 nuclear intensity did not changed by inhibitors. Although, treatment with α-amanitin declined the nucleoplasmic transcription, the concomitance of nascent RNAs and CDK9 accumulations was evident around the NLB, and a rim of nascent transcripts surrounding the nucleolus was detectable reproducibly in these oocytes which was essentially lacking in their ActD-treated counterparts. These results indicated that both nuclear and nucleolar transcription were sensitive to CDK9 inhibition.

### CDK9 activity is necessary for proper rRNA transcription in GV oocytes

**Fig 4:**
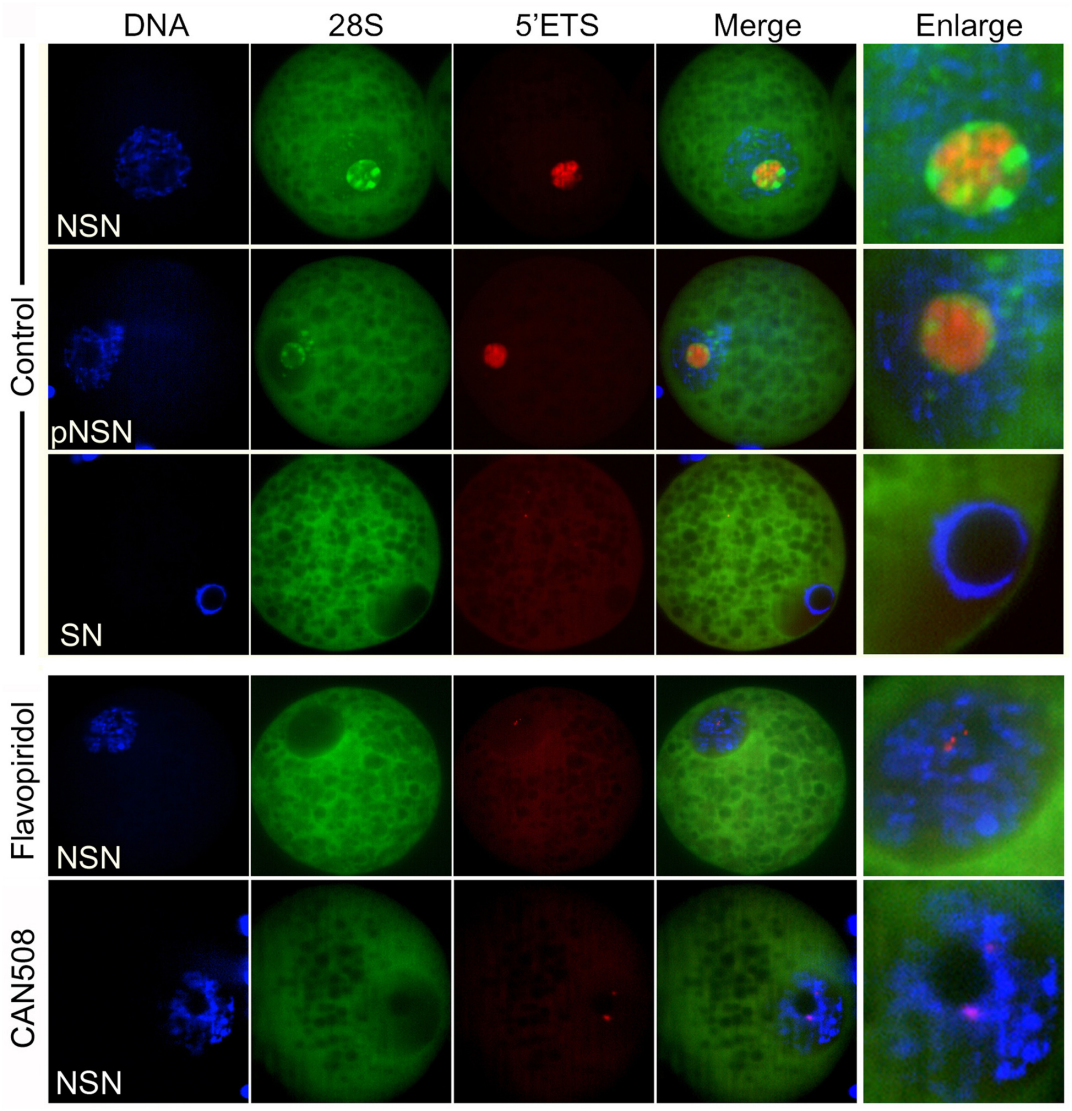
CDK9 kinase activity is necessary for proper rRNA transcription in GV oocytes. COCs were obtained from different size of antral follicles and immediately were treated or not with Flavopiridol or CAN508 for 1 hour in complete maturation medium. Then, they were denuded, fixed and subjected to RNA-FISH. Two fluorophore-conjugated DNA oligonucleotides were designed to probe for two distant regions of pre-rRNAs. One Cy3-conjugated probe recognized the 5’ external transcribed spacer (5’ETS, red) and the other, FAM-conjugated probe recognized the 28S rRNA (green) at the 3’ end of the pre-rRNA. In control group, the level and localization of 5’ETS and 28S in oocytes with different chromatin configuration was analyzed. While in NSN oocytes, signals from both probes were robust and centralized inside the NLB, in SN oocytes no signals were detected from either probe. Inhibition of CDK9 by Flavopiridol or by CAN508 essentially abolished the signals from both probes in the majority of transcriptionally active oocytes.

Because FU labeling of nascent RNAs does not distinguish between RNA polymerases’ products, we set out to revisit the effect of CDK9 inhibition on oocyte nucleolar transcription by applying rRNA-FISH. To this end, fluorescently labeled DNA probes were designed to detect two distant parts of pre-rRNA; one probe complementary to 34 nucleotides of the 5’ external transcribed spacer (5’ETS), and another probe complementary to 34 nucleotides of 28S rRNA. Since we found no report of similar study in pig oocytes, before the analysis of CDK9 inhibition, we first collected all the oocytes of different follicular size to analyze the situation of pre-RNA transcription and processing in these cells. We found that in NSN oocytes, a strong central signal emitted from 5’ETS surrounded by mutually exclusive localization of 28S rRNA probe. In pNSN oocytes the state of 5’ETS remained almost unchanged but 28S RNA became weaker in its intensity and marginal in its localization (Fig 4). As expected SN oocytes exhibited no signal from either probe. As such, defuse 28S signal was observed throughout the rest of the oocyte in all chromatin configurations. In both cases, no FISH signals were observed when sense probes to 5’ETS or 28S were used (data not shown). Then we analyzed the effect of CDK9 inhibition on pre-rRNA level in NSN or pNSN oocytes. COCs were treated or not for 1 hour with Flavopiridol and then were denuded and subjected to rRNA-FISH. In treated oocytes, nucleolar signals corresponding to both probes were essentially abolished in ~92% of the NSN and all the pNSN oocytes, and the defused cytoplasmic signal of 28S remained unchanged. Similarly, treatment with CAN508 also decreased both rRNA signals. These results indicate that inhibition of CDK9 activity either by Flavopiridol or by CAN508, even for a short time, disrupted proper rRNA transcription.

### CDK9 activity is required for cumulus cell expansion and nuclear maturation in oocytes

**Fig 5:**
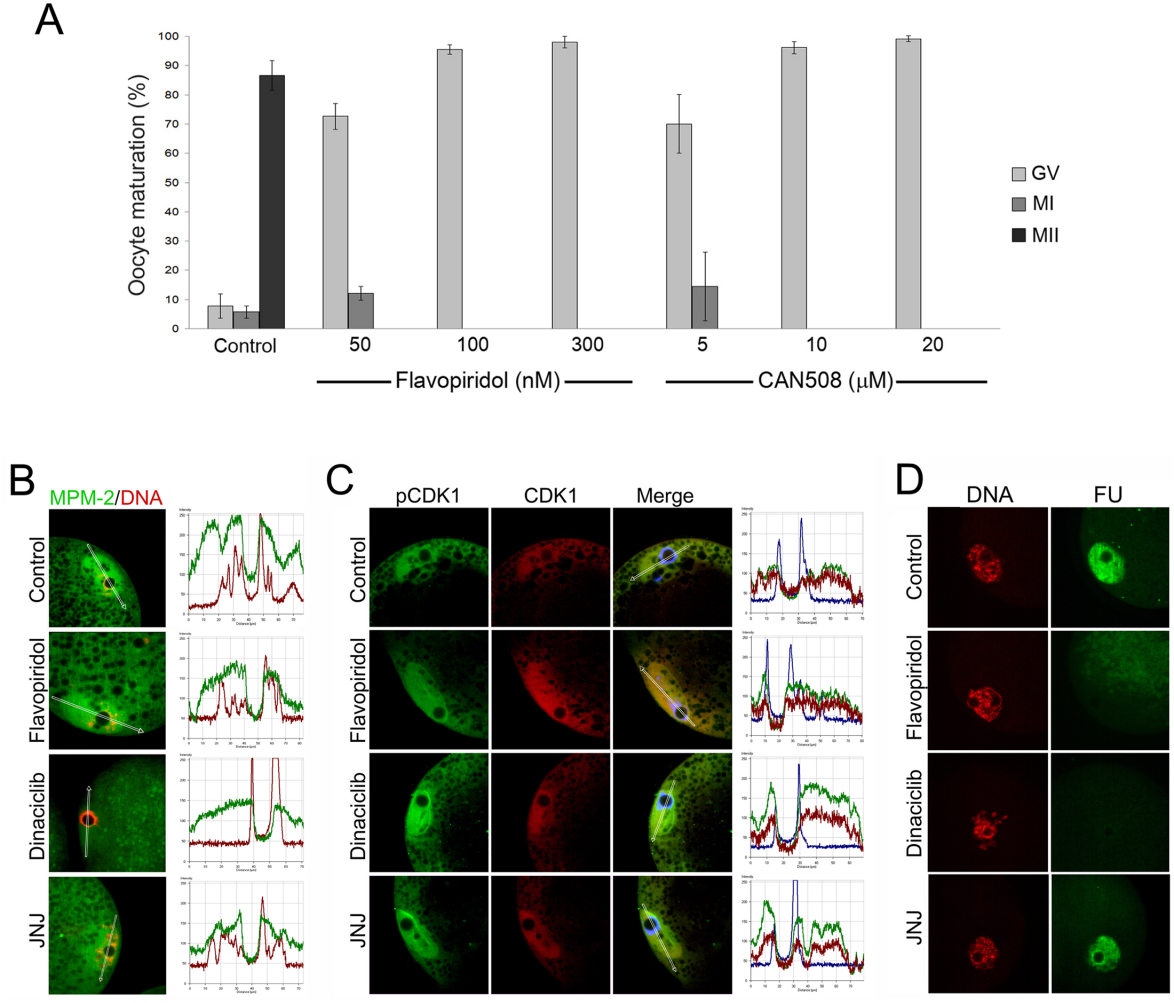
Effect of CDK9 inhibition on oocyte maturation and the MPF kinase activity. **A)** COCs were obtained from small and medium size antral follicles and matured in absence or presence of increasing concentration of Flavopiridol or CAN508. Then COCs were denuded and fixed and subjected to immunostaining against nuclear lamin A/C. DNA counterstained with DAPI. Oocytes with two distinct metaphase chromosomes and polar body were considered as MII. Oocytes with only one metaphase chromosome and no polar body were considered as MI. Oocytes with intact germinal vesicle and positive lamin A/C staining were considered as GV. The figure shows that inhibition of CDK9 strictly inhibits GVBD in pig oocytes. **B)** COCs were obtained from large antral follicles and treated or not with CDK inhibitors, Flavopiridol, Dinaciclib and JNJ-7706621 for 6 hours in complete maturation medium. Then, COCs were denuded and subjected to immunostaining. MPF/CDK1 kinase activity was measured using a monoclonal antibody recognizing the proteins that share two peptide motives LTPLK and FTPLQ. CDK1 phosphorylates the Thr residue of these motives and promotes GVBD. MPM-2 (green) recognizes these motives only when they are phosphorylated. Although treatment with Flavopiridol blocked GVBD, but the level of MPM-2 signal did not changed significantly compared with untreated oocytes. On the other hand, both Dinaciclib and JNJ-7706621 (JNJ) decline the level of CDK1 kinase activity. **C)** CDK1 activity is inhibited by phosphorylation of Thr14/Tyr15 residues. Dephosphorylation of these residues activates CDK1 and promotes GVBD. In Flavopiridol-treated oocytes, the level of pCDK1 did not change compared with the control group, but Dinaciclib, and less potently JNJ, elevated the level of inhibitory phosphorylation of CDK1. In all groups, the level of pCDK1 was normalized with pan CDK1. **D)** COCs were obtained from small or medium size antral follicles. Inhibition of CDK9 by Flavopiridol or Dinaciclib inhibited transcriptional activity of GV oocytes, but inhibition of CDK1 by JNJ did not changed the level of nascent RNAs compared with untreated oocytes. These experiments totally showed that GVBD block by Flavopiridol was due to inhibition of transcription but not inhibition of CDK1 activity.

Although we previously showed that inhibition of CDK9 kinase activity blocked mouse embryo development beyond the 2-cell stage, to the best of our knowledge, the effects of this inhibition on any other mammalian oocyte or embryo have never been examined. The experiments described in this study showed the inhibition of CDK9 essentially suppressed the transcriptional activity of the oocytes. However, it was not known if this inhibitory action of the CDK9 inhibitors had any effect on nuclear maturation and resumption of meiosis in these cells. Because Flavopiridol showed the most potent inhibitory on CDK9 in nanomolar scale, we first examined the effect of different concentrations of Flavopiridol on COCs cultured *in vitro* up to 44 hours. First, we treated COCs with 50, 100 or 300 nM Flavopiridol for 22 hours and monitored the expansion of cumulus cells. Measurements under a light microscope equipped with a graded optical lens revealed that by 100 nM or higher concentrations, the expansion of cumulus cells was dramatically declined compared to non-treated control group (S1 Fig). Then, COCs were withdrawn from gonadotropins and cultured for more 22 hours in the same concentrations of Flavopiridol and examined under the microscope. The results showed that even at concentration as low as 100 nM, Flavopiridol reduced the cumulus cell expansion dramatically. Then, we analyzed the effect of CDK9 inhibition on the nuclear maturation of pig oocytes. COCs were cultured in maturation medium as described above without or with 50, 100 and 300 nM Flavopiridol or with 5, 10 and 20 μM CAN508 and then subjected to immunostaining. A monoclonal antibody against nuclear lamin A/C was used to examine the status of germinal vesicles. This experiment showed that in pig oocytes, GVBD is inhibited in 95.5% of the oocytes treated with 100 nM Flavopiridol and in 96% of the oocytes treated with 10 μM of CAN508 (Fig 5A). No chromatin deformity or degeneration of oocytes was observed during the period of culture in presence of Flavopiridol.

Based on the results mentioned above, we chose 100 nM concentration of Flavopiridol and 10 μM of CAN508 as the minimal effective concentrations of the compounds for further experiments. Since GVBD was inhibited by CDK9 inhibition, we asked whether the inhibitory effect of Flavopiridol was, at least in part, due to inhibition of MPF (CDK1/Cyclin B) activity in GV oocytes. To address this question, we set to analyze the effect of CDK9 inhibition on both CDK1 kinase activity and inhibitory phosphorylation of CDK1 on Thr14/Tyr15 residues by immunocytochemistry. Low activity of CDK1is required for meiotic prophase I arrest, which resembles the G2 phase of mitosis in somatic cells [36]. Increased kinase activity of CDK1 promotes GVBD and resumption of meiosis (G2-M transition). Activation of CDK1 triggers a cascade of phosphorylation of numerous proteins many of them sharing two peptide motives LTPLK and FTPLQ [37]. A monoclonal antibody, MPM-2, can recognize these motives when they are phosphorylated on the Thr residue. Initially, we observed that treatment of COCs with 250 nM of a potent CDK1 inhibitor, Dinaciclib (SCH727965) for 6 hours decreased the level of MPM-2 fluorescence intensity in GV oocytes. But, since CDK9 also is inhibited by Dinaciclib, we chose another CDK inhibitor, JNJ-7706621 which inhibits specifically CDK1 but not CDK9. This compound also decreased the level of MPM-2 by 250 nM but less potently than Dinaciclib.

Then, we repeated the experiment to compare the effects of these two CDK inhibitors with that of Flavopiridol. COCs were cultured in a complete maturation medium in absence or presence of compounds for 6 hours and then subjected to immunostaining against MPM-2 antigens. Analysis of the specimens by confocal microscopy revealed that in untreated oocytes, the fluorescence intensity of MPM-2 was only slightly higher than that of the oocytes in which CDK9 activity was inhibited by Flavopiridol (Fig 5B). In Dinaciclib treated oocytes, the level of MPM-2 signal significantly reduced compared to control or Flavopiridol-treated oocytes. Treatment with JNJ-7706621 also significantly reduced the level of MPM-2 but less potently. Treatment with these inhibitors showed that, for a relatively short time, MPM-2 level is sensitive to CDK1 inhibition but not CDK9 inhibition. Then, we analyzed oocytes in the same conditions as described above for the phosphorylation status of CDK1 on Thr14/Tyr15 residues using an antibody specifically recognizing these residues only when both of them are phosphorylated. When these amino acids are phosphorylated by MYT1 and WEE2 (WEE1B), respectively, the CDK1 kinase activity is inhibited [38]. Confocal microscopy analyses revealed that in the case of Flavopiridol-treated oocytes, the level of inhibitory phosphorylation of CDK1 was not significantly changed compared to that of untreated control group. In both set of experiments the changes of fluorescent intensities was compared with two other CDK inhibitors, Dinaciclib and JNJ-7706621. These CDK1 inhibitors on the other hand, increased the level of CDK1 inhibitory phosphorylation. However, the fluorescence intensity of CDK1 did not significantly change in all the treatments compared to control group (Fig 5C). Also, we tested the effects of these inhibitors on transcriptional activity of the oocytes after 6 hour of treatment. As expected, Flavopiridol and Dinaciclib reduced the signal emitted from the FU labeling to the background level but, the signal remained unchanged in JNJ-7706621-treated oocytes (Fig 5D). These experiments showed that the inhibitory effect of Flavopiridol on oocyte nuclear maturation was the consequence of inhibition of transcription but not inhibition of CDK1 activity. In addition, the experiment showed that inhibition of CDK1 did not affect on transcriptional activity of the oocytes.

### CDK9 activity is essential for embryonic genome activation

**Fig 6:**
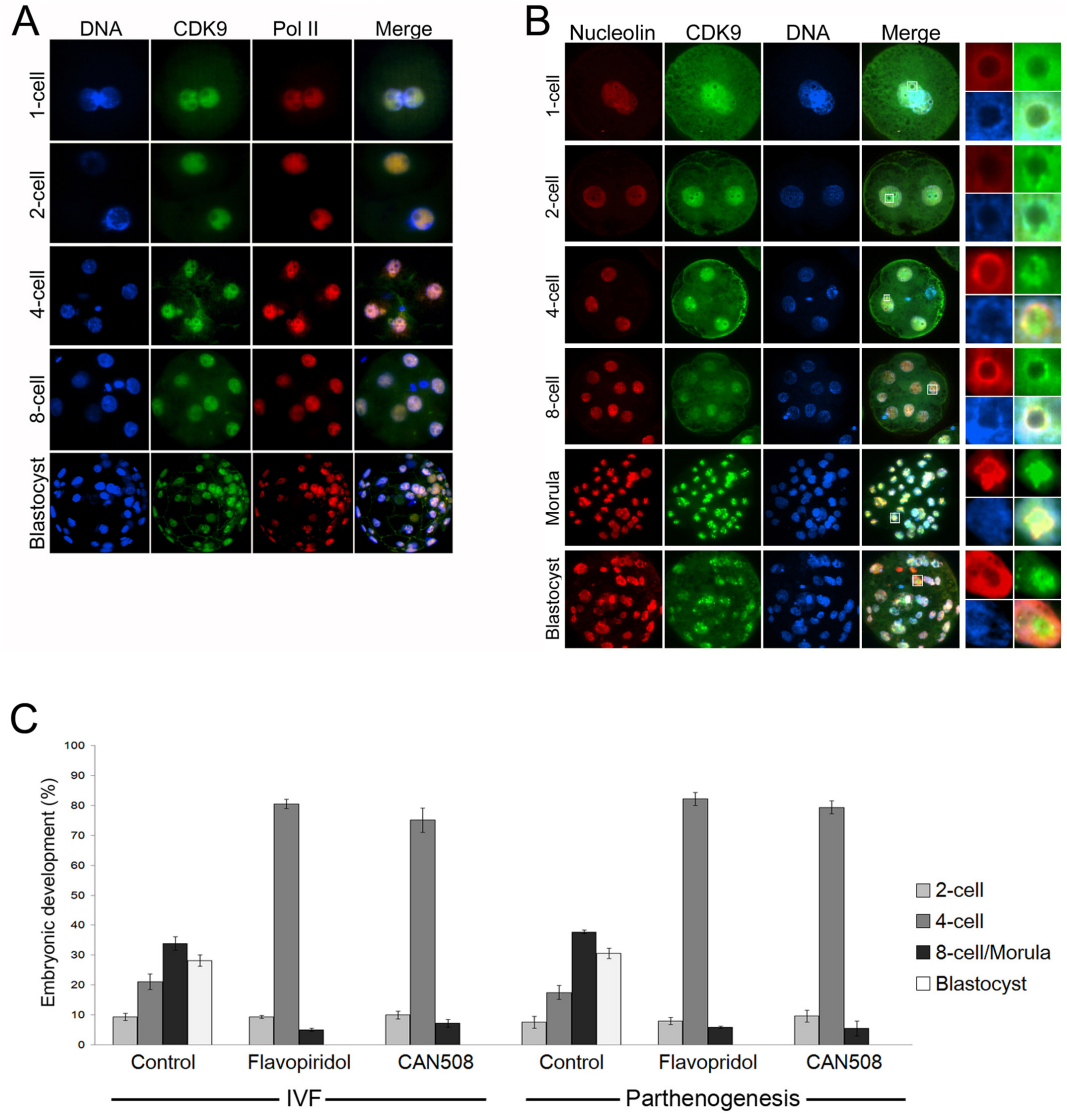
The presence and the necessity of CDK9 in preimplantation embryos. Confocal immunofluorescence analysis of CDK9 in whole-mount IVF and parthenogenetic embryos. **A)** CDK9 nuclear localization in IVF embryos. Pol II (red) was used as the nuclear marker. No intense signal at the periphery of the nucleolar precursor bodies was observed for CDK9. **B)** CDK9 nuclear localization in parthenogenetic embryos. Nucleolin (red) was used as the nucleolar marker. In 1-cell embryos, CDK9 was evenly distributed throughout pronuclei. At the 2-cell stage, CDK9 showed some localization at the periphery of NPBs in some cases. In late 4-cell embryos, CDK9 exhibited more profound nucleolar association and colocalized with nucleolin. As nucleolar precursors transformed to matured nucleoli in morula and blastocysts, CDK9 localized within the nucleoli with and surrounded by nucleolin. DNA was counterstained with DAPI. Enlarged views of the small insets are shown in the right panels. **C)** Embryos were produced via either IVF or parthenogenesis and cultured absence or presence of Flavopiridol (100nM) or CAN508 (10 μM) for 7 days. Then, the embryos were counterstained with DAPI and were analyzed under a fluorescent microscope. Embryos with zero nuclei were excluded. The first cleavage rate was not significantly changed in treated embryos. However, the majority of treated embryos tended to remain at 4-cell stage. Also, progress to next cell cycles was very poor in treated embryos compared with their control groups. No blastocyst was observed in groups treated with either CDK9 inhibitor.

Since the presence and the possible function of P-TEFb in pig early embryos have not been addressed before, we sought to examine the localization of CDK9 in these systems. Also we inhibited the activity of CDK9 by its inhibitors and monitored the consequence events. Pig embryos, produced *in vitro* by *in vitro* fertilization or parthenogenetic activation, were cultured and, at different stages, subjected to immunocytochemical analyses. Initially we observed that in embryos produced by IVF, CDK9 is present in all the stages of preimplantation development and is well colocalized with Pol II (Fig 6A). In majority of the embryos analyzed by confocal microscopy, the nucleolar localization of CDK9 was not evident. Then, we produced parthenogenetic embryos and immunostained CDK9 with the same antibody and observed that in these embryos, CDK9 showed nuclear as well as nucleolar localization at 4-cell stage and onward. Co-immunostainings of CDK9 and nucleolar factor, nucleolin, showed that in the 1-cell stage, CDK9 appeared in the nucleoplasm of both pronuclei and partly colocalized with nucleolin (Fig 6B). A stronger CDK9 signal, at the periphery of nucleolar precursor bodies (NPBs), was observed in the 2-cell stage. A well but still heterogeneous association of CDK9 with NPBs was evident in the late 4-cell stage. The CDK9 signal decreased in intensity in 8-cell embryos, but increased again and continued to be present at matured nucleoli in morula and blastocysts. This indicates that nucleolar association of CDK9 is reduced in presence of male genome, and in parthenogenetic embryos, CDK9 retains its nucleolar association.

Then, we asked whether CDK9 kinase activity is necessary for embryo development. Embryos, produced via IVF or parthenogenesis, were treated with 100 nM Flavopiridol for 7 days. At the end of culture, all the embryos were stained with DAPI and analyzed under a fluorescent microscope. Degenerated embryos or embryos with zero nuclei were excluded from analyses. Also, the experiment was repeated with 10 μM CAN508. Treatment with either compound strictly inhibited the embryo development beyond the 4-cell stage (Fig 6C). Although the rate of the first cleavage did not change in treated embryos compared to their untreated counterparts, the majority of both IVF and parthenogenetic embryos stopped developmental progress beyond the 4-cell stage in presence of CDK9 inhibitors. The rates of formation of 8-cell/morula embryos were 33.8% and 37.2% in IVF and parthenotes respectively. These rates were significantly decreased in embryos treated with either compound, and no blastocyst formation was observed in inhibitory conditions.

### CDK9 activity is necessary for proper embryo transcription

**Fig 7:**
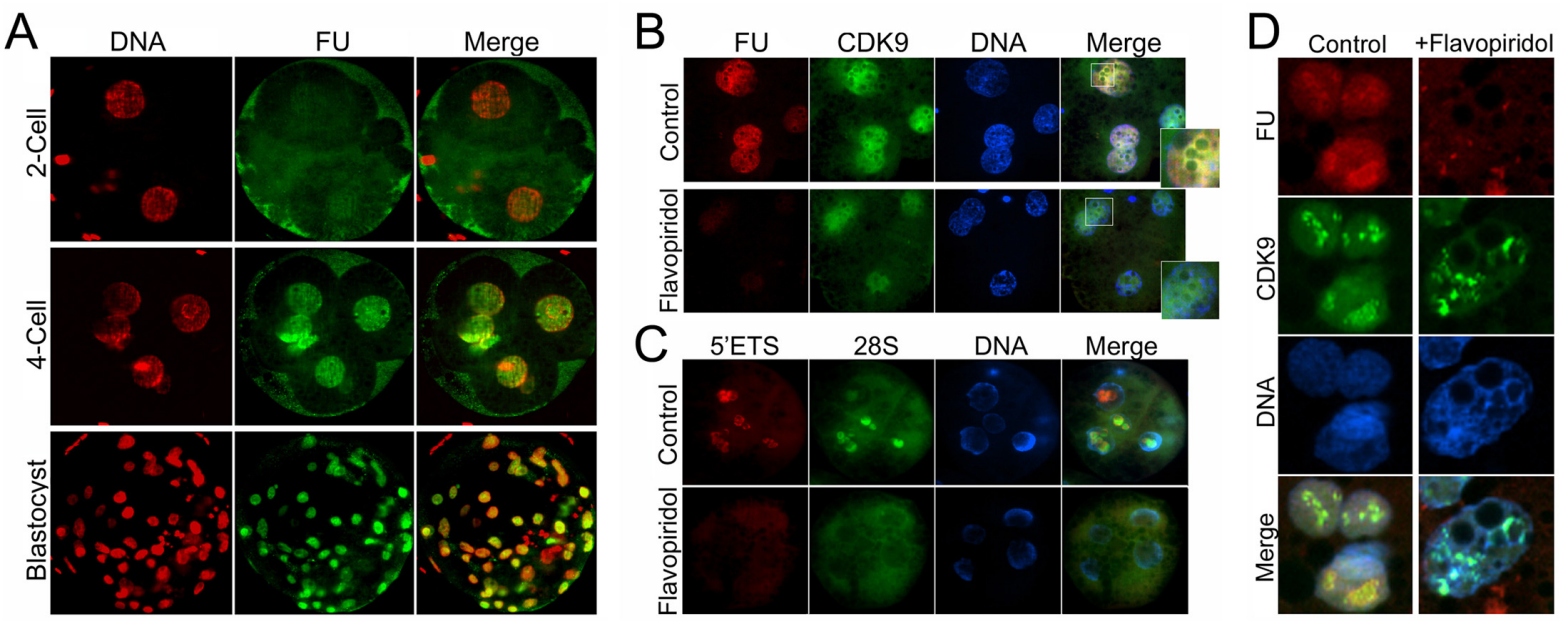
Effect of CDK9 inhibition on transcriptional activity of preimplantation embryos. **A)** The susceptibility of FU labeling was determined in preimplantation embryos. Embryos were cultured in PZM-3 medium. For nascent RNA labeling, embryos were transferred to the same medium plus 5mM FU and cultured for additional 1 hour. Then embryos were fixed and subjected to immunostaining. While 2-cell embryos showed very faint, if any, nascent RNA level, 4-cell stage embryos and blastocysts exhibited strong nuclear signals from FU incorporation into nascent RNAs. **B)** IVF embryos were cultured in PZM-3 medium in presence or absence of 100 nM Flavopiridol. One hour before fixation, embryos were transferred to the same media supplemented with FU. CDK9 inhibition dramatically decreased the level of nascent RNAs in late 4-cell stage embryos. The level of nuclear CDK9 also decreased slightly in these embryos. **C)** Treatment of late 4-cell embryos for 1 hour with Flavopiridol abolished the pre-rRNA transcription. Although the signal corresponding to both 5’ETS and 28S rRNA was evident in the center of NPBs in most blastomeres’nuclei of untreated control embryos, such signals were not observed in their treated counterparts. **D)** Blastocysts were treated or not with Flavopiridol for 1 hour and simultaneously were labeled with FU. Then embryos were fixed and co-immunostained against FU and CDK9. In control group, the transcriptional activity was at higher level in the areas with less DAPI staining and corresponding to nucleoli. CDK9 also predominated in these areas. In treated embryos, both nucleoplasmic and nucleolar nascent RNAs were declined. Also nucleolar localization of CDK9 was unraveled. Only individual cells from the whole blastocysts are shown.

Then we asked whether inhibition of CDK9 affects the transcriptional activity of the embryos. First, we took advantage of FU labeling of nascent RNAs in IVF embryos and found that this method can be applied in pig early embryos. Labeling of nascent RNAs revealed that at 2-cell stage, embryos show very faint signal of labeled RNAs, but the signal significantly increased at 4-cell stage and blastocysts (Fig 7A). Next, we cultured embryos in presence or absence of Flavopiridol up to late 4-cell stage followed by FU treatment for 1 hour. Then, embryos were fixed and immunostained against FU. Also CDK9 was co-immunostained to evaluate the nuclear situation of the kinase in its inhibitory condition. In the majority of untreated late 4-cell stage embryos, with four distinct round and prominent DAPI-stained nuclei, signals corresponding to the FU labeling were predominated in nuclei and enriched around the NPBs definitely coinciding with the signals from CDK9 immunostaining (Fig 7B). In treated embryos, on the other hand, the level of transcription was essentially abolished compared with untreated group. Also, the intensity of the nascent RNAs surrounding the NPBs was negatively affected. The level of CDK9 also was slightly weaker than that of the untreated embryos.

We then were curious about the potential role of CDK9 in rRNA transcription in preimplantation embryos. To find any relation, 4-cell embryos treated or not with Flavopiridol, were subjected to RNA-FISH using the same probes as indicated in Fig 4. To avoid the secondary effects of a long-term inhibition of CDK9 that may affect the synthesis of proteins involved in rRNA transcription, we treated late 4-cell stage embryos only for 1 hour. In this stage, although in some blastomeres only a faint signal was emitted from either rRNA, we detected both 5’ETS and 28S rRNA accumulations in the center of NPBs in most blatomeres of almost all the untreated embryos. On the other hand, such accumulations were not evident in treated embryos (Fig 7C). This indicated that CDK9 kinase activity is crucial fro proper rRNA production in 4-cell stage embryos. Transcriptional activity also was globally suppressed in blastocysts treated with Flavopiridol for 1 hour. FU labeling detected nascent RNAs in untreated blastocyst nuclei and nucleoli but such labeling was not detectable in treated embryos. Also nucleolar accumulations of CDK9 were unraveled in these embryos (Fig 7D). Collectively, these experiments showed that the main reason for the developmental arrest in embryos, in which the CDK9 activity was inhibited, was the severe deficiency in activation of their embryonic genome.

## Discussion

The presence and the function of P-TEFb components have never been investigated in porcine oocyte maturation and embryo development. The present study provides a new insight into a possibility by which P-TEFb kinase activity contributes to both the regulation of the oocyte maturation and to genome activation of preimplantation embryos. The presence of CDK9 and Cyclin T1 in splicing-rich nuclear speckle regions has already been shown in earlier studies [39, 40]. As such, it has been shown that phosphorylated CDK9 on Thr186 (pCDK9, active form of CDK9) is present in nuclear speckles, and that neither Thr186 phosphorylation nor enzymatic activity of CDK9 is required for its localization to nuclear speckles [40]. Previous findings indicated that inhibition of general transcription results in formation of larger speckles due to accumulation of factors residing in speckle regions [41]. Also, our observations support at least the role of P-TEFb in general transcription in porcine oocytes. However, this study suggests another exciting role for P-TEFb at least in porcine early development. Both CDK9 and Cyclin T1, and to some extent pCDK9, showed colocalization with nascent RNAs surrounding the nucleolus-like bodies. In our previous effort to detect active sites of transcription in GV oocytes, a ring-shaped and relatively intense accumulation of nascent RNAs can be observed around the NLBs which are resistant to low concentrations of α-amanitin [6]. Such ring-shaped nascent RNAs also have been observed in mouse NSN oocytes [5]. This nascent RNAs are generally attributed to Pol I transcriptional activity. Here, we observed that CDK9 colocalized with these RNAs suggesting a possible involvement of this kinase in Pol I transcription (Fig 2). CDK9 and Cyclin T1 also showed a very interesting association to nucleolar factors in pig GV oocytes. CDK9 showed a better colocalization with UBF. However, a partial co-localization of CDK9 with another nucleolar factor, fibrillarin, was evident. UBF, a member of the Pol I transcription initiation complex, has been shown to overlap partly with the rRNA processing territories containing fibrillarin [42]. This suggests that CDK9 may be involved in rRNA transcription or rRNA processing in these cells.

The presence and the localization of some accumulations of CDK9 closed to the oocyte nucleoli provide highly suggestive speculation that CDK9 participates in nucleolar function in these cells. Comparison between oocytes treated with transcription inhibitors (ActD and α-amanitin) with different mechanism of action revealed that both Pol I- and Pol II-dependent transcriptions are sensitive to CDK9 activity. To understand the potential role of CDK9 in Pol I-dependent transcription, we inhibited the enzyme’s kinase activity using a CDK9 specific inhibitors, Flavopiridol and CAN508 (Fig 3). Flavopiridol inhibits CDK9 in a non-ATP competitive manner in sub-micromolar concentrations *in vitro* and in vivo [24, 43, 44]. Other CDKs such as CDK1 also show sensitivity to this drug but in concentrations higher than 1μM [24, 45]. In present study we used Flavopiridol at the minimal concentration we previously found to inhibit EGA in mouse i.e. 100 nM. Also, this concentration has been reported the minimal concentration to effectively inhibit CDK9 *in vivo* in other studies [45–49]. When the effect of Flavopiridol on the transcriptional activity of GV oocytes were compared to that of α-amanitin or ActD, it was revealed that inhibition of CDK9 abrogated the nuclear transcription almost globally, similar to the action of ActD. In oocytes treated with either compounds, the nucleoplasmic nascent RNAs were declined; but these oocytes did not show a rim of nascent RNA remaining around the NLBs which was observed reproducibly in oocytes treated with α-amanitin. This indicates that in addition to Pol II transcription, Pol I transcription also were sensitive to CDK9 inhibition.

Labeling of nascent RNAs with a cell permeable halogenated nucleotide, FU, overcomes the difficulty of BrUTP application that should mostly be injected into oocytes or embryos. However, these nucleotide analogues do not distinguish between the transcripts of different RNA polymerases. To address the potential role of CDK9 specifically in Pol I transcription, we applied fluorescently labeled probes to detect pre-rRNA transcripts. We could not find any similar report in the literature addressing the situation of rRNA transcription in pig oocytes or embryos, so we applied a standard RNA-FISH procedure with some modifications (Fig 4). The probes were complementary to two distant parts of pre-rRNA. One probe detected an initial part of the pre-RNA, 5’ETS, which is short-lived transcript and is processed rapidly during rRNA transcription. The expression of 5’ETS reflects the rate of Pol I transcription initiation [50]. The second probe detected 28S rRNA which persists after being processed and is transported to cytoplasm to engage with ribosome machinery. In this study, we found that in NSN oocytes, 5’ETS was centrally accumulated within the NLBs. 28S also showed a centrally positive signal but with different localization with 5’ETS. In some parts of NLB, accumulations of 28S and 5’ETS were evident which were mutually exclusive. These sites reflect the different parts of the NLB in which rRNA production and rRNA processing occur. Recently, it has been shown that in mouse NSN oocytes, rRNA transcripts can be detected inside the NLBs only when the oocytes are fixed with 70% ethanol [51]. Our modified protocol resulted in a very clear illustration of rRNA accumulations inside the NLBs. Labeling rRNAs also showed that by oocyte growth, 28S and to some extent 5’ETS become more marginalized to the surface of the NLB. This is in accordance with the previous finding indicating a gradual marginalizing of FCs in growing pig oocytes [52]. On the other hand, inhibition of CDK9 for a short time (1 hour), decreased the level of both 5’ETS and 28S in almost all NSN and pNSN oocytes. Burger et al. (2010) have shown that short-time treatment of human cells with Flavopiridol impairs early rRNA processing [53]. When cells were treated with 800 nM Flavopiridol, the production of 32S, 18S and 28S rRNAs decreased dramatically. Also recently, the authors showed that blocking CDK9, either by small molecule inhibitors (Flavopiridol, DRB, Cdk9-inhibitor II/CAN508 and KM05283SC) or by RNAi impairs pre-rRNA processing which in turn, feeds back on Pol I transcription and alters the kinetics of nascent 47S rRNA accumulation [54]. The role of CDK9 in rRNA transcription seems to be conserved. Ctk1 is a yeast well-characterized kinase closed to human CDK9, which in addition to its role as Pol II CTD kinase, is partially localized to the nucleolus, interacts with Pol I and plays role in rRNA transcription [55]. Accordingly, disruption of either Ctk1 or Bur1 (CDK9 homologue in yeast) impairs rRNA processing [54, 56]. Several other factors known to be involved in Pol II transcription have also reported to be involved in Pol I transcription in yeast and higher eukaryotes [57–61]. c-Myc/Max heterodimer, for example, binds to and recruits P-TEFb complex to Myc targets [62]. C-Myc also is directly involved in rRNA processing [50, 63–65]. In current study, we observed that CDK9 colocalizes with c-Myc at the periphery of NLBs, highly suggesting that CDK9 might be involved in rRNA processing via recruitment by c-Myc (data not shown). The identity of the CDK9 substrate (s) in the nucleoli remains to be elucidated. However, this study suggests an indispensible role for this kinase in ribosome biogenesis in pig oocytes.

Also we showed that CDK9 function is crucial for resumption of meiosis in pig oocytes (Fig 5A). It is well established that in presence of transcription inhibitors, oocytes of large animals fail to accomplish their GVBD. For example, bovine oocytes are arrested at GVBD in presence of α-amanitin [66] or DRB [67]. Also pig oocytes are unable to undergo GVBD in presence of α-amanitin [68]. As such, inhibition of protein synthesis by cycloheximide blocks GVBD in both species [66, 69, 70]. Thus, it seems that both protein synthesis and mRNA transcription are required for oocytes to progress beyond the GV stage. It is generally accepted that Flavopiridol inhibits P-TEFb and blocks most mRNA transcription [24, 43]. Also, it has been shown that CDK9 is possibly involved in mRNA translation. Either chemical inhibition or shRNA knockdown of CDK9 results in decline in eIF4E phosphorylation on its Ser209 which in turn, reduces the translation of mRNAs [71]. Therefore, it is likely that inhibition of CDK9 blocks GVBD via inhibition of both gene transcription and protein synthesis.

The second most sensitive CDK to Flavopiridol is CDK1 [24, 72]. CDK1 kinase activity is necessary for mouse GVBD [73]. Our unpublished data show that by 100 nM, Flavopiridol does not inhibit mouse GVBD *in vitro*. Our analyses showed that Flavopiridol did not inhibit CDK1 kinase activity, but both Dinaciclib and JNJ-7706621 negatively affected the level of phosphorylation of CDK1 targets. Also, treatment with JNJ-7706621 confirmed that CDK1 inhibition blocked GVBD in a transcription-independent manner.

We previously have shown that CDK9 inhibition blocks mouse embryos at 2-cell stage *in vitro* [74]. We also were curious whether CDK9 activity is necessary for proper embryo development in pig. We found that in inhibitory conditions, a great proportion of pig embryos failed to progress beyond 4-cell stage (Fig 6C). It is well established that in pig embryos, genome activation occurs at 4-cell stage [75, 76]. Therefore, CDK9 is a kinase necessary for EGA in pig. It seems that the role of CDK9 in EGA is conserved, since inhibition of CDK9 by RNAi leads to early embryonic lethality in *C*. *elegans* [33] and Drosophila [34]. In both species, embryonic death occurs at mid-blastula transition, which is the time of EGA in these animals. As expected, inhibition of CDK9 did not alter the nuclear localization of CDK9 but abolished transcription in arrested embryos (Fig 7B), indicating that many genes whose expression are necessary for EGA are essentially regulated by CDK9 activity. Also we tried to understand the potential role of CDK9 in nucleolar transcription in pig embryos. Our dual immunostaining revealed that nucleolar association of CDK9 was prominent in pig parthenogenetic embryos (Fig 7B). In these embryos, CDK9 colocalized with nucleolin from the third cell cycle which is the time of EGA in this species. At the end of this cell cycle, nucleoli become recognizable [77] and the rRNA transcription commences [78, 79]. In latter reference, authors reported the activation of rDNAs heterogeneously at late 4-cell stage. However, the probe used in their study only recognized genomic ribosomal DNA but not its transcript. In our study, we could detect both 5’ETS and 28S rRNAs in 4-cell stage embryos as clear in GV stage oocytes (Figs 4 and 7C). Only in some blastomeres signals emitted from rRNAs were faint which is in accordance with the heterogeneous activation of genomic rDNAs. In blastocysts on the other hand, mature nucleoli are formed. Nascent RNA labeling in this stage gave a clear accumulation of positive FU signals inside nucleoli. This signals as well as nucleoplasmic signals could not be detected in short-time (1 hour) Flavopiridol-treated embryos indicating the sensitivity of both nuclear and nucleolar RNA transcripts to CDK9 inhibition (Fig 7D).

In conclusion, our data show the importance of CDK9 kinase activity in both resumption of meiosis and embryonic genome activation in pig. Moreover, we find that CDK9 is involved in both Pol I- and Pol II-dependent transcription in pig early development.

## Supporting Information

S1 Fig Effect of Flavopiridol on cumulus cell expansion *in vitro*. COCs were collected from large antral follicles and were cultured in complete maturation medium with gonadotropins in presence or absence of 100 nM Flavopiridol. After 22 hours, the expansion of cumulus cells was assessed under a graded light microscope and then, COCs were transferred to the same medium without gonadotropins and analyzed 22 hours later. The figure shows that Flavopiridol strictly inhibited the cumulus cell expansion in pig COCs. **A, B** and **C)** treated COCs and **A’, B’** and **C’)** untreated COCs assessed after 0, 22 and 44 hours, respectively. **D)** Mean diameters of COCs in both group at indicated times of culture.
(DOCX)
