# Supplementary material for: P-TEFb Kinase Activity Is Essential for Global Transcription, Resumption of Meiosis and Embryonic Genome Activation in Pig
Source: PLoS One. 2016 Mar 24;11(3):e0152254. doi: 10.1371/journal.pone.0152254 (PMC4807088; doi:10.1371/journal.pone.0152254)
Supplement: S1 Fig — COCs were collected from large antral follicles and were cultured in complete maturation medium with gonadotropins in presence or absence of 100 nM Flavopiridol. After 22 hours, the expansion of cumulus cells was assessed under a graded light microscope and then, COCs were transferred to the same medium without gonadotropins and analyzed 22 hours later. The figure shows that Flavopiridol strictly inhibited the cumulus cell expansion in pig COCs. A, B and C) treated COCs and A’, B’ and C’) untreated COCs assessed after 0, 22 and 44 hours, respectively. D) Mean diameters of COCs in both group at indicated times of culture. (DOCX) [file pone.0152254.s001.docx]

**Supporting Information**

**S1 Fig.** **Effect of Flavopiridol on cumulus cell expansion *in vitro*.** COCs were collected from large antral follicles and were cultured in complete maturation medium with gonadotropins in presence or absence of 100 nM Flavopiridol. After 22 hours, the expansion of cumulus cells was assessed under a graded light microscope and then, COCs were transferred to the same medium without gonadotropins and analyzed 22 hours later. The figure shows that Flavopiridol strictly inhibited the cumulus cell expansion in pig COCs. **A, B** and **C)** treated COCs and **A’, B’** and **C’)** untreated COCs assessed after 0, 22 and 44 hours, respectively. **D)** Mean diameters of COCs in both group at indicated times of culture.

**S1 Fig**

**
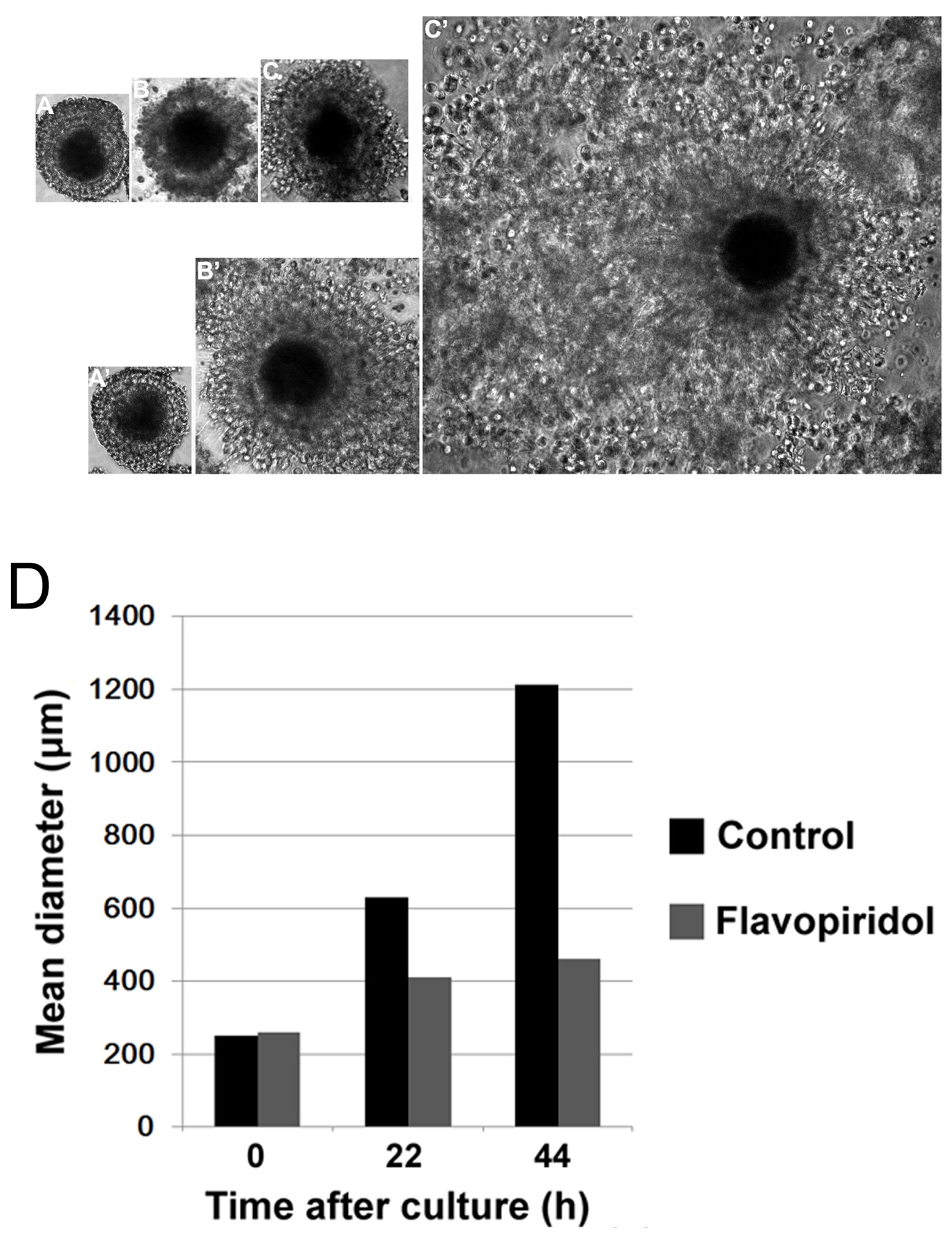
**
